# A high-throughput chemically induced inflammation assay in zebrafish

**DOI:** 10.1186/1741-7007-8-151

**Published:** 2010-12-22

**Authors:** Claudia A d'Alençon, Oscar A Peña, Christine Wittmann, Viviana E Gallardo, Rebecca A Jones, Felix Loosli, Urban Liebel, Clemens Grabher, Miguel L Allende

**Affiliations:** 1 Center for Genome Regulation, Facultad de Ciencias, Universidad de Chile, Santiago, Chile; 2 Departamento de Ciencias Biologicas, Facultad de Ciencias Biologicas, Universidad Andres Bello, Santiago, Chile; 3 Institute of Toxicology and Genetics, Karlsruhe Institute of Technology, Hermann von Helmholtz Platz 1, D-76344 Eggenstein-Leopoldshafen, Germany; 4 Department of Biochemistry, School of Medical Sciences, University of Bristol, Bristol BS2 8HD, UK

## Abstract

**Background:**

Studies on innate immunity have benefited from the introduction of zebrafish as a model system. Transgenic fish expressing fluorescent proteins in leukocyte populations allow direct, quantitative visualization of an inflammatory response *in vivo*. It has been proposed that this animal model can be used for high-throughput screens aimed at the identification of novel immunomodulatory lead compounds. However, current assays require invasive manipulation of fish individually, thus preventing high-content screening.

**Results:**

Here we show that specific, noninvasive damage to lateral line neuromast cells can induce a robust acute inflammatory response. Exposure of fish larvae to sublethal concentrations of copper sulfate selectively damages the sensory hair cell population inducing infiltration of leukocytes to neuromasts within 20 minutes. Inflammation can be assayed in real time using transgenic fish expressing fluorescent proteins in leukocytes or by histochemical assays in fixed larvae. We demonstrate the usefulness of this method for chemical and genetic screens to detect the effect of immunomodulatory compounds and mutations affecting the leukocyte response. Moreover, we transformed the assay into a high-throughput screening method by using a customized automated imaging and processing system that quantifies the magnitude of the inflammatory reaction.

**Conclusions:**

This approach allows rapid screening of thousands of compounds or mutagenized zebrafish for effects on inflammation and enables the identification of novel players in the regulation of innate immunity and potential lead compounds toward new immunomodulatory therapies. We have called this method the chemically induced inflammation assay, or ChIn assay.

See Commentary article: http://www.biomedcentral.com/1741-7007/8/148.

## Background

Inflammation is a reaction of the immune system to tissue damage and infection and represents a key component in normal tissue homeostasis. Consequently, deregulated inflammatory reactions result in severely detrimental chronic conditions. One of the hallmarks of the innate inflammatory response is infiltration of the affected tissue by leukocytes of the innate immune system (that is, granulocytes and macrophages). Inflammatory cells are recruited to the site of wounding or infection by proinflammatory mediators such as hydrogen peroxide, cytokines and chemokines [1]. Studying the molecular and cellular basis of inflammation *in vivo *is often hampered by the opacity of the tissue, and, to date, most studies have relied on *in vitro *assays or on analysis after tissue fixation. Given the transparency of the zebrafish during early developmental stages, the availability of transgenic fluorescent reporter lines and the conservation of the principal components of the innate immune system, it is now possible to study immunity by following the behavior of infiltrating cells in the living animal. Green fluorescent protein (GFP)-labeled leukocytes can be observed in larval or adult models of inflammation, which involve wounding or exposing fish to infectious agents. In this animal model, it was recently discovered that hydrogen peroxide is an important immediate signaling molecule required for the rapid recruitment of leukocytes to wounds [2]. Given the simplicity with which these assays can be carried out in the zebrafish and a limitless and cheap supply of animals, it has been proposed as a useful system for high-throughput small-molecule screens aimed at detecting immunomodulatory activity *in vivo *[3-6] or for genetic screens aimed at identifying key molecular components of the innate immune response [7-9]. The small size of fish larvae offers the added advantage of testing candidate molecules by directly dissolving them in small volumes of fish water; usually dimethyl sulfoxide (DMSO) is added as a solvent to allow penetration of the compounds to all tissues. Previous reports have proposed laser damage directed to the yolk surface of embryos [10] and localized nicks in fin tissue or amputation of the entire tail fin in larvae [3,5,6] to induce inflammation. These treatments are one of the bottlenecks for performing large-scale screens, as the animals have to be manipulated individually prior to distribution into microtiter well plates for phenotypic analysis.

We have been studying the induction of cell death and regeneration in neuromasts of the lateral line system of zebrafish larvae. Neuromasts, small clusters of mechanosensory hair cells enclosed within a compact group of accessory cells, are regularly distributed over the body surface and can be damaged by exposure to physical or chemical insults [11]. We have found that copper sulfate added to the incubation medium rapidly induces cell death in neuromasts [12,13], though they are able to rapidly regenerate and reach full functionality 1 day after the damaging agent is removed [14]. In the present work, we have discovered that damage to neuromasts is followed immediately by migration of cells that express high levels of *matrix metalloproteinase 9 *(*mmp9*), a marker of myeloid lineage cell populations [15]. Using transgenic lines that label myeloid leukocytes (neutrophils and/or macrophages) *in vivo*, we observed a specific, extremely rapid and highly reproducible innate immune response to copper-induced neuromast damage. Since the wounds are localized and are elicited chemically, no invasive manipulation of fish is required and the treatment can be applied massively. Exploiting this observation, we developed a quantitative measure for inflammation by counting leukocytes migrating to the lateral line neuromasts in transgenic lines or with immune cell-specific stains upon copper-induced neuromast damage. We further tested the method by using known anti-inflammatory drugs, and we demonstrate detection of their activity as they potently inhibit leukocyte infiltration of the neuromasts. Resolution of the response can also be scored if drugs are added after damage. Furthermore, analysis of fish that are mutant for the *Wiskott-Aldrich syndrome *(*was*) gene [7] exemplifies the power of this approach for recovering mutations in genes involved in leukocyte migratory behavior. Finally, we show that the procedure can be massively scaled up by automation of distribution of individual larvae in microtiter wells, liquid handling, image acquisition and quantification of the inflammatory response in real time. Thus, we introduce a new method for high-throughput screens aimed at detecting immunomodulatory activity of small molecules. We anticipate that chemically induced inflammation assays (ChIn) will make it possible to achieve different types of high-throughput compound screens as well as genetic screens, focusing on aspects of the wound-induced inflammatory response (initiation and resolution), analyses of various types of infection-induced responses, investigation of tissue regeneration and specific cell subtype migration assays.

## Results

### Myeloid leukocytes migrate to damaged neuromasts

Zebrafish larvae establish the primary lateral line system by 3 days postfertilization (dpf). Addition of copper sulfate to the water rapidly destroys hair cells of the lateral line system by inducing oxidative stress followed by cell death [12,13]. Among genes induced in larvae by copper exposure, we detected strong stimulation of the *mmp9 *gene (VEG, OAP and MLA, unpublished work). When we carried out *in situ *hybridization to detect *mmp9 *transcripts in control animals, we detected very low levels of expression in a few cells located within the posterior blood island (PBI) or caudal hematopoietic tissue (CHT), the areas where most myeloid leukocytes reside at this developmental stage. However, the same analysis carried out in copper-exposed larvae showed strong speckled signals in discrete clusters along the flanks of the trunk and tail, a distribution that suggested that immune cells expressing *mmp9 *actively migrate toward the damaged neuromasts (Additional file 1).

To visualize the presence of leukocytes of the innate immune system and to follow their behavior *in vivo *after copper treatment, we used fish carrying the myeloperoxidase or lysozyme C promoters driving the expression of GFP or red fluorescent protein in myeloid leukocytes (most likely neutrophils), *BACmpx::GFP *and *lysC::DsRED2*, respectively [5,6]. Both transgenic lines produced identical results in our assays.

**Figure 1:**
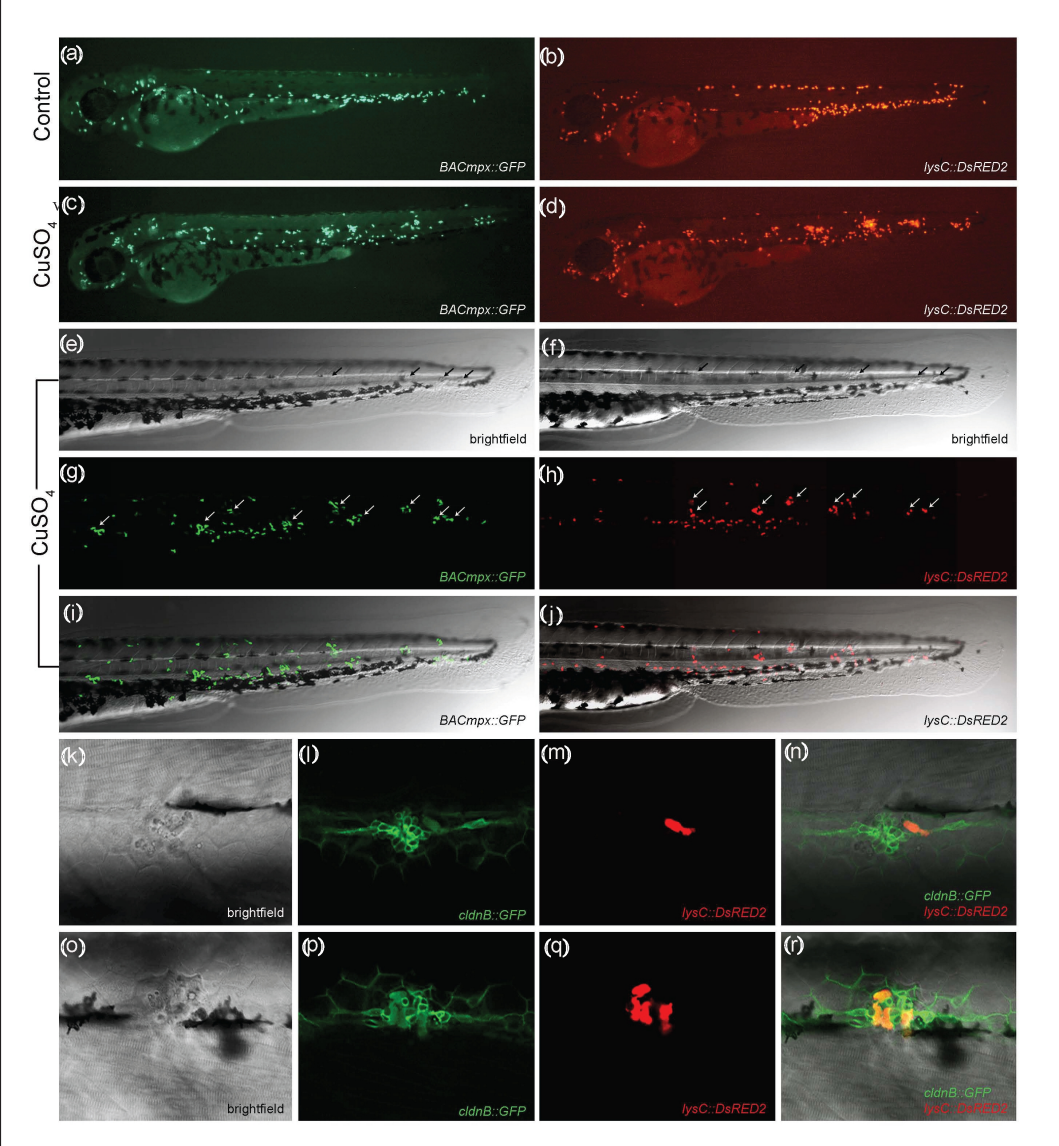
**Leukocytes migrate specifically to damaged lateral line neuromasts in zebrafish larvae**. **(a-j) **56-hours postfertilization (56-hpf) *BACmpx::GFP *or *lysC::DsRED2 *transgenic zebrafish larvae exhibit green or red fluorescent leukocytes, respectively. **(a **and **b) **Untreated fish show the normal distribution of labeled cells, mostly localized in the ventral trunk and tail. **(c **and **d) **In copper-treated siblings, leukocytes become localized preferentially to a few clusters along the horizontal midline of the trunk and tail. **(e-j) **A detailed view of this region in copper-treated animals shows that while many cells disperse throughout the body, other cells congregate in discrete clusters (arrows); no overt tissue damage to the larvae is observed in bright-field images. **(k-r) **A mating cross of *cldnB::GFP *and *lysC::DsRED2 *transgenic fish labels neuromasts in green and leukocytes in red. Posterior trunk neuromasts were imaged immediately after adding copper **(k-n) **or 20 minutes after copper treatment **(o-r) **using bright-field red or green fluorescence illumination. Few, if any, leukocytes are seen near neuromasts at the beginning of treatment. **(m **and **n) **Here a case where a single leukocyte is present is shown. **(q **and **r) **In contrast, copper-treated fish have numerous red fluorescent leukocytes interspersed within the neuromast cells. Note the extent of damage induced by copper in the neuromast cells (compare Figures 1l and 1p).

We exposed transgenic zebrafish larvae at 56 hours postfertilization (hpf) to 10 μM CuSO_4 _for a period of 2 hours and monitored the behavior of fluorescent leukocytes immediately after beginning the treatment. In control (untreated) fish, most of the immune cells remained in the PBI or CHT (Figures 1a and 1b). In contrast, examination of zebrafish larvae exposed to copper showed a general dispersal of these cells, suggestive of active migration from their initial location. Most striking was the rapid coalescence of labeled cells to form regularly spaced clusters in the midline of the trunk and tail of treated animals (compare Figures 1a and 1b with Figures 1c and 1d). This cell clustering can be observed for up to 3 hours after removal of copper from the medium (Figures 1e-1j). We next confirmed that migratory immune cells home specifically toward damaged neuromasts. We generated compound transgenic fish by mating the *lysC::DsRed *transgenic zebrafish line, labeling leukocytes in red [6], with the *cldnB::GFP *transgenic zebrafish line, in which neuromast cells are labeled green [16]. In these double-labeled fish, we observed that clustering of leukocytes occurs specifically around neuromasts that have suffered damage (Figures 1k-1r). Whereas in the absence of copper treatment immune cells patrol the area near the neuromasts only occasionally, in treated fish numerous immune cells concentrate in neuromasts and remain in their vicinity for several hours. Similar results have been obtained with red fluorescent or vital dye-labeled neuromasts and GFP-labeled leukocytes (not shown).

To examine the dynamics of the inflammatory process under these conditions, we captured time-lapse images of the trunk and tail of *BACmpx::GFP *fish beginning immediately after the addition of copper (see Additional file 2). The cells began to respond to the damage around 15 minutes after the addition of copper to the medium, and the first labeled cells reached the neuromasts at around 20 minutes. In the presence of copper sulfate, leukocytes remained in the neuromast area, maintaining a circulatory patrolling movement within it for 2-3 hours, after which they began to disperse and no longer concentrated exclusively near neuromasts. Six hours after the removal of copper, treated larvae were indistinguishable from controls, indicating resolution of inflammation.

Neuromasts consist of centrally located hair cells surrounded by mantle cells on the surface and supporting cells at the base, all forming a compact rosette [11]. After the addition of copper, the arrival of macrophages, neutrophils and possibly other immune cells coincided with a progressive disruption of the rosette-like structure (Figures 1k-1r and Additional file 3). This disruption is likely caused by a combination of cell death [12,13] and the invasion of immune cells, which continuously traverse the interstitial space between neuromast cells, separating them from one another.

The two transgenic lines used in this study label myeloid leukocytes, most likely neutrophils. However, up to 48 hpf, the *lysC::DsRED2 *line labels early macrophages in addition to neutrophils. To learn whether the cell populations labeled in both lines showed similar dynamics in their response, we used a *lysC::DsRED2*/*BACmpx::GFP *compound transgenic line. At 48-56 hpf, most labeled cells expressed both transgenes, but some cells labeled only with DsRED2. However, both double-labeled cells and DsRED2-labeled cells migrated toward the damaged neuromasts (Additional file 4). Thus, it is likely that both neutrophils and macrophages participate in the inflammatory response elicited by copper in neuromasts, and the deciphering of specific roles for either population in real time would require the availability of additional subpopulation-specific transgenic reporter lines.

To determine whether copper-induced damage in other tissues also stimulated an inflammatory response, we exposed *lysC::DsRED2 *zebrafish larvae permanently for up to 7 days with 10 μM CuSO_4_. Prolonged exposure resulted in a general dispersal of leukocytes compared to controls and, in addition to lateral line neuromasts, leukocyte infiltration was observed in the gills and nose (Additional file 5). We conclude that waterborne exposure of larvae to copper sulfate causes lesions to superficial tissues followed by specific inflammatory responses at the sites of damage.

### Quantification of immune cell infiltration in the lateral line after chemical damage

**Figure 2:**
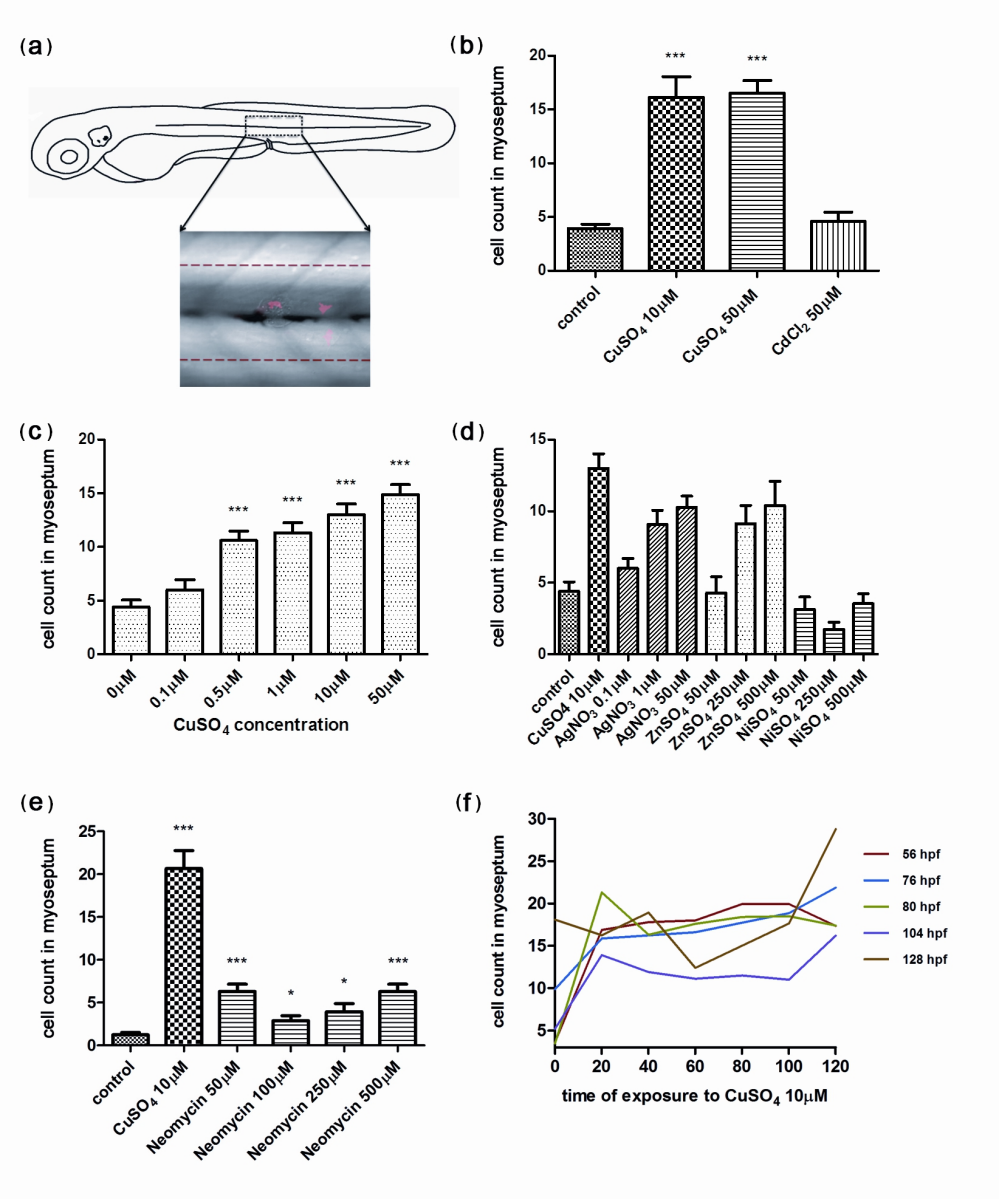
**Quantification of infiltrating leukocytes in the lateral line after diverse treatments: the chemically induced inflammation (ChIn) assay**. **(a) **Schematic view of a 3 days postfertilization (dpf) larva. The boxed area corresponds to the horizontal myoseptum (line). An area of approximately 10 cell diameters is delimited around the myoseptum (dotted red lines) and corresponds to the area where leukocytes were counted in all manual quantification experiments. **(b) **Significant induction of leukocyte recruitment to the lateral line by copper treatment. The graph shows average leukocyte numbers in the lateral line in negative controls (untreated fish or cadmium chloride-treated fish) and in copper-treated fish. **(c) **The effect of copper on leukocyte recruitment to the lateral line is concentration dependent. **(d) **Effectiveness of other metals in the ChIn assay. **(e) **Neomycin, at concentrations that eliminate hair cells, also induces leukocyte recruitment, but less effectively than copper. **(f) **Larvae of different ages, from 56 to 128 hpf, were exposed to 10 μM CuSO_4 _and were monitored for leukocytes present at the myoseptum every 20 minutes thereafter until 120 minutes. Fish at all stages analyzed showed similar behaviors and exhibited increased presence of leukocytes at the lateral line by 20 minutes after initiating exposure to copper. For all experiments, at least 15 larvae were used for each condition. ***P < 0.001.

Taking advantage of our findings, we aimed to develop a simple but robust quantitative method to measure the degree of leukocyte infiltration in damaged neuromasts. Using such a protocol would allow us to reveal the effect of molecules that modulate the inflammatory response elicited by copper exposure, opening the door for chemical or genetic high-throughput inflammation screens. Our first approach (manual quantification) requires only visual inspection of treated and control larvae and can thus be carried out using low-power magnification. It is necessary to have a method for detection of innate immune cells; the optimal way is to use transgenic lines such as *BACmpx::GFP*, *lysC::GFP *or *lysC::DsRED2*, though we have successfully used Sudan Black (Additional file 6) and diaminobenzidine (not shown) staining to label leukocytes histochemically, with identical results in all cases. For quantification, it is not critical to label the neuromasts as the primary lateral line is always localized along the horizontal myoseptum and two or three neuromasts are predictably located above the dorsal aorta, posterior to the cloaca, at the larval stages used (56-72 hpf). Two hours after addition of copper sulfate to the water, immune cells reproducibly and robustly congregate near the neuromasts. Given the highly dynamic nature of the immune cells' behavior, we found it most convenient to fix the larvae at this time to examine all individuals at a similar stage in the response. The GFP or DsRED2 label remains visible for at least 1 day after fixation, allowing sufficient time for quantification of a large sample of larvae. Larvae were observed under a fluorescence dissecting scope, and we established an arbitrary area of approximately five cell diameters above and below the horizontal myoseptum, which runs from the first somite to the end of the tail (Figure 2a). Fluorescent cells were counted within this area on one side of 15 larvae for each treatment, and averages were calculated. Control fish were either untreated or incubated in DMSO (the solvent used when fish were treated with drugs; see below, in the next section of Results). We first designed an experiment using two concentrations of copper (10 μM and 50 μM CuSO_4_) that are toxic to neuromast cells after 2 hours of exposure, and one of cadmium chloride (50 μM CdCl_2_), which causes no damage to neuromast cells at that concentration and exposure time [13]. Quantification by two independent observers showed that there was a significant difference in the number of leukocytes localized to the lateral line in copper-treated fish compared to control (untreated or cadmium-exposed) fish (Figure 2b). This experiment provides proof of principle that a quantitative inflammation assay can be carried out using copper-induced damage and leukocyte cell counts in the lateral line. We next carried out a series of experiments to explore variations of the method that would yield improved results. We first determined that the inflammatory effect of copper sulfate is concentration dependent and that significant leukocyte infiltration can be seen beginning at 0.5 μM CuSO4 (Figure 2c). Of other metals known to affect the viability of neuromast hair cells, only silver, albeit less effectively, yielded significant infiltration at concentrations comparable to copper. Zinc requires a 25-fold higher concentration to result in a significant effect, while nickel was ineffective at the evaluated dosages (Figure 2d). We had previously determined that these other metals have additional toxic effects and that fish survival is compromised [13]. Therefore, we find that copper sulfate remains the most effective and reproducible damaging agent for neuromasts. Interestingly, neomycin, a potent ototoxic aminoglycoside antibiotic known to ablate hair cells in zebrafish neuromasts [17], generated a significant but modest inflammatory response (Figure 2e). We confirmed that hair cells are eliminated in our neomycin treatments by using a hair cell-specific transgenic line (Additional file 7). Whether this fourfold difference reflects a specific property of metal vs. antibiotic-induced damage or whether neomycin somehow affects immune cell migration remains to be determined.

As treatment with 10 μM copper for up to 2 hours does not affect the viability of the larvae, we used this concentration of metal as the inflammation-inducing dose for all subsequent experiments. To determine the optimal developmental stage at which to carry out the inflammation assays and the times at which to obtain the best response after treatment, we exposed fish of different ages (56 hpf to 128 hpf) and performed immune cell counts at the myoseptum between 20 and 120 minutes after the addition of copper. All ages tested showed comparable response curves, whereas significant infiltration of the lateral line by leukocytes was always detected by 20-40 minutes after initiating copper treatment (Figure 2f). However, fish at ages from 56 hpf to 80 hpf yielded slightly more robust responses. We thus established our basic protocol using 56-hpf larvae that spontaneously hatched, incubating the larvae for 40 minutes in 10 μM copper, followed by fixation and evaluation of leukocyte infiltration into the myoseptum (see Methods). We suggest a 40-minute incubation with copper only as a matter of convenience, as there is no significant difference from a 2-hour long incubation. We have called this method the chemically induced inflammation, or ChIn, assay.

### Effect of anti-inflammatory drugs and inhibition of reactive oxygen species

**Figure 3:**
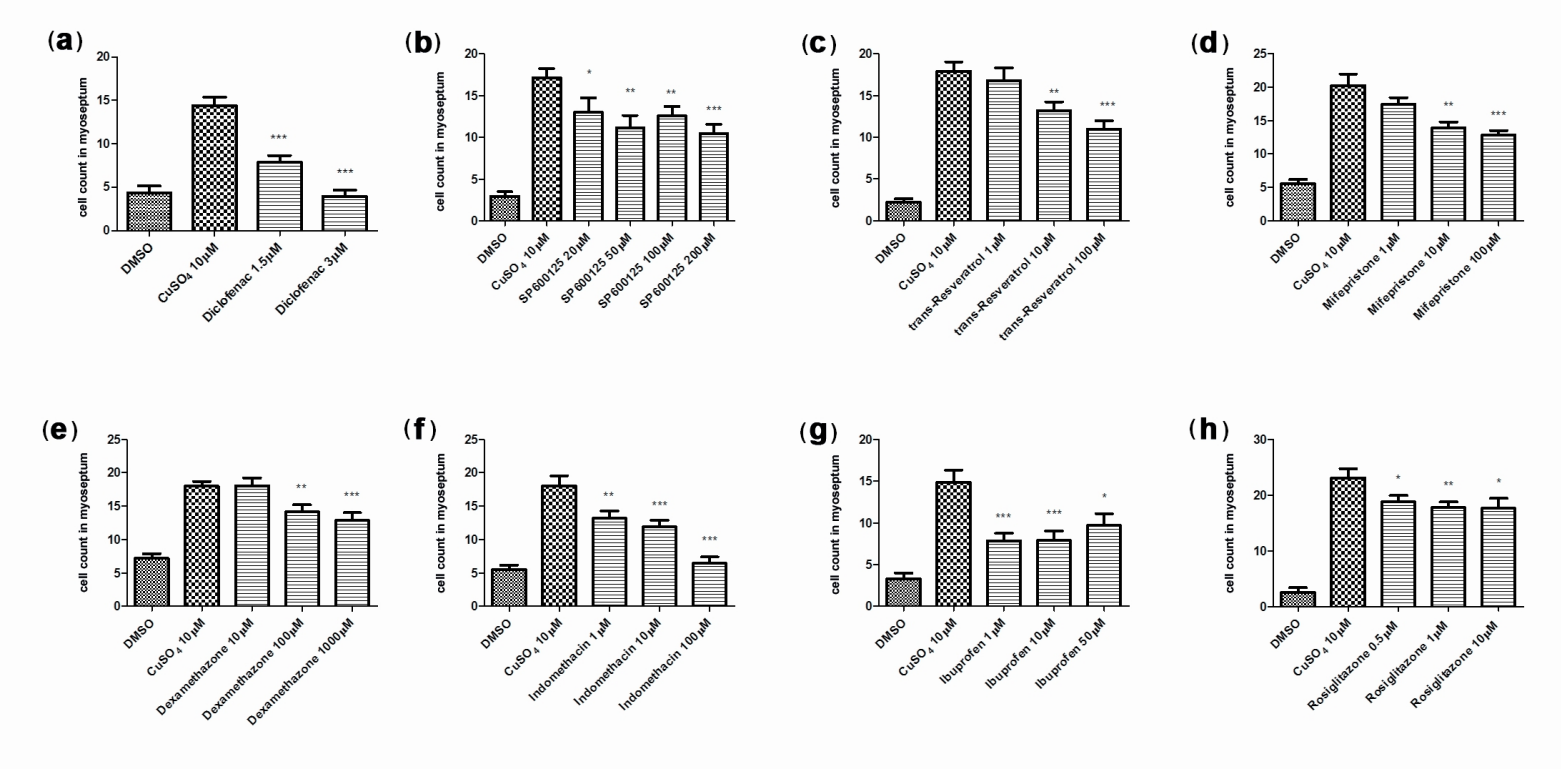
**Proof of principle of the ChIn assay using known anti-inflammatory drugs**. The ChIn assays were carried out by manually counting leukocytes recruited to the lateral line after copper treatment (10 μM) as described in Results. In this experiment, dimethyl sulfoxide (DMSO) was included in all samples, and drugs were added 1 hour prior to copper treatment at the indicated concentrations. As before, experiments were carried out with 15 larvae per condition. ***P < 0.001. **0.001 <*P *< 0.01. *0.01 <*P *< 0.05.

Previous reports have shown that well-established anti-inflammatory drugs behave as predicted in zebrafish inflammation assays that rely on physical wounding [3,18,19]. We wanted to test whether the ChIn assay is also able to detect the activity of these and other molecules that are known to act at different points during inflammation. Selected compounds (see Table 1) were added to fish medium containing 1% DMSO 30 minutes to 1 hour prior to the addition of copper, allowing for effective drug penetration of larval tissues. Of the 11 drugs tested using the manual ChIn assay, 10 of them exhibited statistically significant inhibition of leukocyte infiltration at varying concentrations ranging from 0.5 μM to 100 μM (Table 1). For a large-scale, small-molecule screen, we would thus recommend use of a two-concentration standard assay (10 μM/100 μM). For the panel of compounds tested in this study, such an experimental setup would identify more than 90% of effective compounds. Only hydrocortisone required a higher concentration (300 μM) to yield significant inhibition of inflammation. A quantitative analysis for a subset of the drugs tested in the manual ChIn assay is shown in Figure 3.

**Table 1:** Detection of anti-inflammatory activity using the ChIN assaya

Compound	Mode of action	Concentration (μM)	Significant effect	Reference
Ibuprofen	COX inhibitor	1	***	[38]
		10	***	
		50	*	
Diclofenac	COX inhibitor	1.5	***	[38,39]
		3	***	
SP600125	JNK inhibitor	20	*	[40]
		50	**	
		100	**	
		200	***	
*Trans*-resveratrol	COX-1 inhibitor	1	-	[41]
		10	**	
		100	***	
Mifepristone (RU486)	Progesterone and GR antagonist	1	-	[42]
		10	**	
		100	***	
Dexamethasone	Steroidal nitric oxide synthase inhibitor	10	-	[43]
		100	**	
		1,000	***	
Indomethacin	COX inhibitor	1	**	[39]
		10	***	
		100	***	
Rosiglitazone	PPAR-γ agonist	0.5	*	[44]
		1	**	
		10	*	
Aspirin	COX inhibitor	10	-	[39]
		20	**	
Hydrocortisone	Steroidal GR agonist	1	-	[45]
		10	-	
		100	-	
		300	*	
Sulindac	NS COX-1 inhibitor	1	**	[46]
		10	***	
		50	***	
		100	***	

^a^Selected drugs were added to the incubation medium 1 hour prior to addition of copper and were tested at the indicated concentrations for inhibition of leukocyte migration using *BACmpx::GFP *larvae in chemically induced inflammation assays (ChIn) (10 μM CuSO_4 _for 40 minutes). Up to four concentrations were chosen to provide an overview of drug activity in the ChIn assay. We aimed at identifying a concentration yielding significant results with *P *< 0.001. In some instances, this was not possible (rosiglitazone, aspirin, hydrocortisone) as higher concentrations were lethal or showed reduced significance compared to lower concentrations. All drugs were used in medium containing 1% dimethyl sulfoxide, as were control fish. Asterisks indicate significant leukocyte migration inhibition. ***P < 0.001. **0.001 <*P *< 0.01. *0.01 <*P *< 0.05. Minus sign indicates no significant difference. GR, glucocorticoid receptor; NS, nonsteroidal; COX, cyclooxygenase; JNK, c-Jun N-terminal kinase; PPAR-γ, peroxisome proliferator-activated receptor-γ.

**Figure 4:**
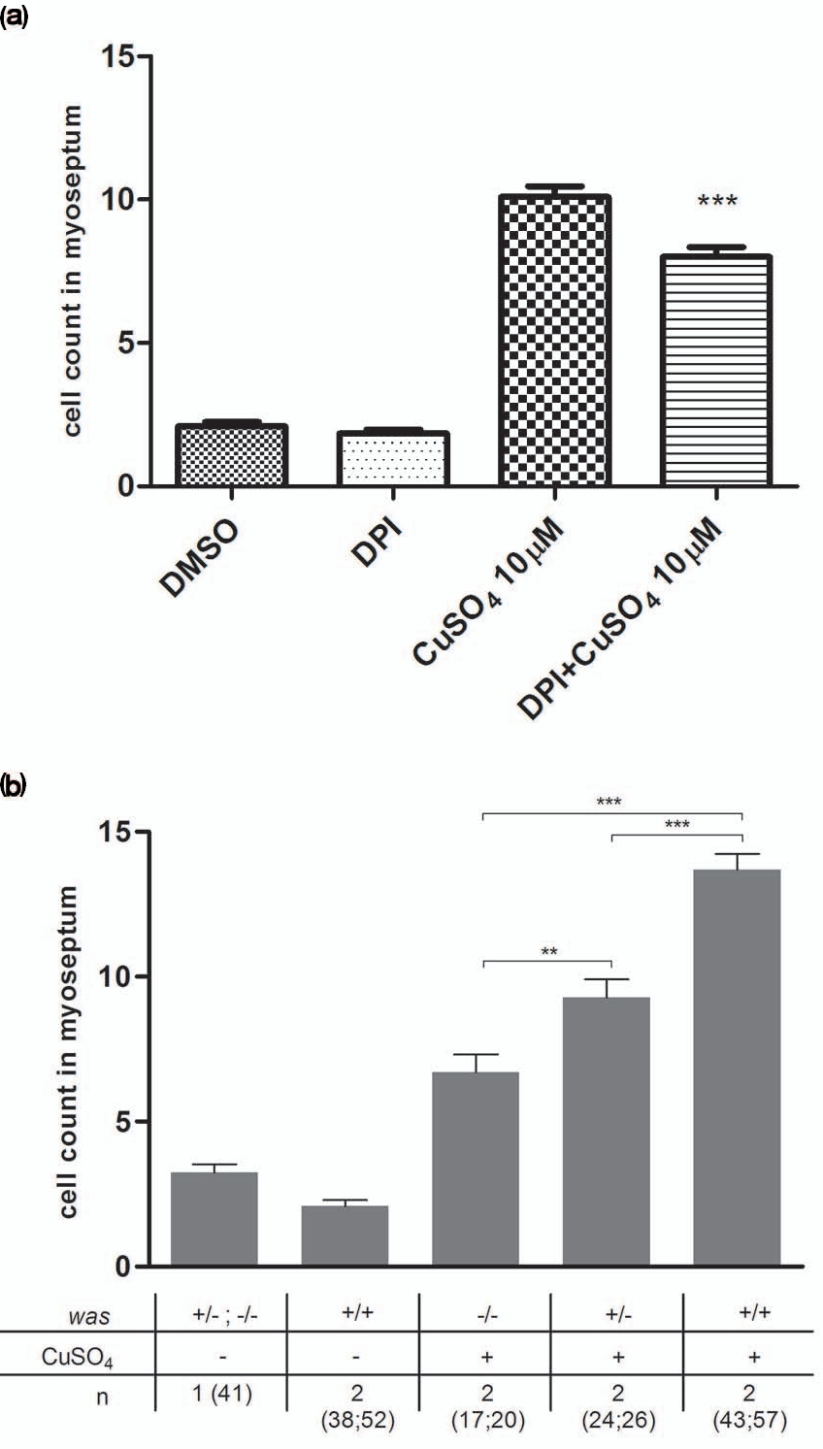
**Detection of H_2_O_2 _inhibition and genetic mutations using ChIn assays**. **(a) **Reactive oxygen species signaling is critical for copper-induced inflammation. Diphenyleneiodonium (DPI), an inhibitor of NADPH oxidases, impairs wounds to leukocyte signaling, resulting in reduced leukocyte numbers at damaged neuromasts. Larvae at age 56 hpf were treated with 100 μM DPI for 1 hour prior to copper treatment (10 μM CuSO_4 _for 40 minutes). DPI treatment results in significantly lower leukocyte numbers within the myoseptum compared to untreated control larvae. **(b) **The ChIn assay enables detection of genetic mutations affecting an inflammatory response. Clutches from matings between homozygous *Wiskott-Aldrich syndrome *(*was*) gene males and heterozygous *was *females were treated with 10 μM CuSO_4_, fixed and scored by Sudan Black staining, and individual larvae were subsequently genotyped. Both homozygous and heterozygous *was *mutants recruited significantly lower numbers of leukocytes to the myoseptum than wild-type larvae upon copper-induced neuromast damage. In addition, the inflammatory response of homozygous mutants is significantly lower than that of heterozygous mutants. **P = 0.0045. ***P < 0.001.

To further assess the benefit of the ChIn assay for detecting novel mechanisms guiding inflammation, we were curious whether we would be able to identify the recently described role of reactive oxygen species (ROS) in this process. Formation of a gradient of H_2_O_2 _has been shown to be required for leukocyte recruitment toward wounds inflicted by tailfin transection in zebrafish [2]. To evaluate whether copper-induced wounding also involves ROS signaling and whether such a role can be detected using the ChIn assay, we tested the NADPH oxidase inhibitor diphenyleneiodonium (DPI) using the ChIn assay (Figure 4a). Indeed, pretreatment of larvae with DPI significantly reduced the number of leukocytes infiltrating the lateral line, confirming that ROS gradient formation is also critical for leukocytes to respond to copper-mediated lesions and the potential of the ChIn assay to link unexpected molecules to leukocyte infiltration.

As innate immune leukocytes abandon the wounded neuromast a few hours after the response, we next wished to learn whether it would be possible to also detect molecules that affected the resolution phase. Nonsteroidal anti-inflammatory drugs (such as diclofenac and ibuprofen) act as nonselective inhibitors of the enzyme cyclooxygenase (COX), inhibiting both the cyclooxygenase-1 and cyclooxygenase-2 isoenzymes (COX1 and COX2, respectively). COX enzymes are proinflammatory during the early phase of inflammation, but aid resolution at the later phase by generating an alternative set of prostaglandins [20]. Resolution of inflammation was analyzed by carrying out copper damage for 40 minutes as before, adding the drugs immediately after copper removal and scoring for leukocyte infiltration 3 hours after that. Hence, if inflammatory resolution was impaired, we would observe an increase in leukocyte numbers at neuromasts compared to controls. When diclofenac and ibuprofen were added after copper treatment, the number of infiltrating leukocytes after 3 hours was significantly different from control fish, indicating that inflammation was not resolved in these cases (Additional file 8). The number of leukocytes in drug-treated fish was, in fact, almost identical to the number observed immediately after copper removal, suggesting that drug-exposed cells were blocked from exiting the wounded area.

### The ChIn assay can detect mutations that affect the inflammatory response

To explore the full versatility of the ChIn assay, we investigated its potential for detecting genetic mutations within critical pathways of an innate immune response. To this end, we used the zebrafish *was *mutant [7]. The Wiskott-Aldrich syndrome (WAS) family of proteins is involved in transduction of signals from receptors on the cell surface to the actin cytoskeleton. WAS protein deficiency underlies a severe human condition characterized by recurrent infections and autoimmunity caused at least in part by perturbed leukocyte migration toward chemotactic cues [21,22]. We exposed larvae obtained from a cross between homozygous *was *mutant male fish and heterozygous female fish to copper and carried out a ChIn assay; as controls, wild-type fish were used. The sensitivity of the ChIn assay allowed a clear identification of larvae that were either homozygous or heterozygous for the mutation on the basis of infiltration of leukocytes toward neuromasts after copper treatment (Figure 4b). Subsequent genotyping further confirmed our correct phenotypic identification of homozygous mutants from larvae heterozygous for the mutation (not shown). As shown previously, *was *gene dosing correlates with the degree of leukocyte migratory impairment, as heterozygotes show an intermediate effect compared to homozygous mutants and wild-type larvae [7]. The ChIn assay may thus also be used for genetic screens to identify mutations in genes critical for an inflammatory response.

### Automated detection of anti-inflammatory activity

**Figure 5:**
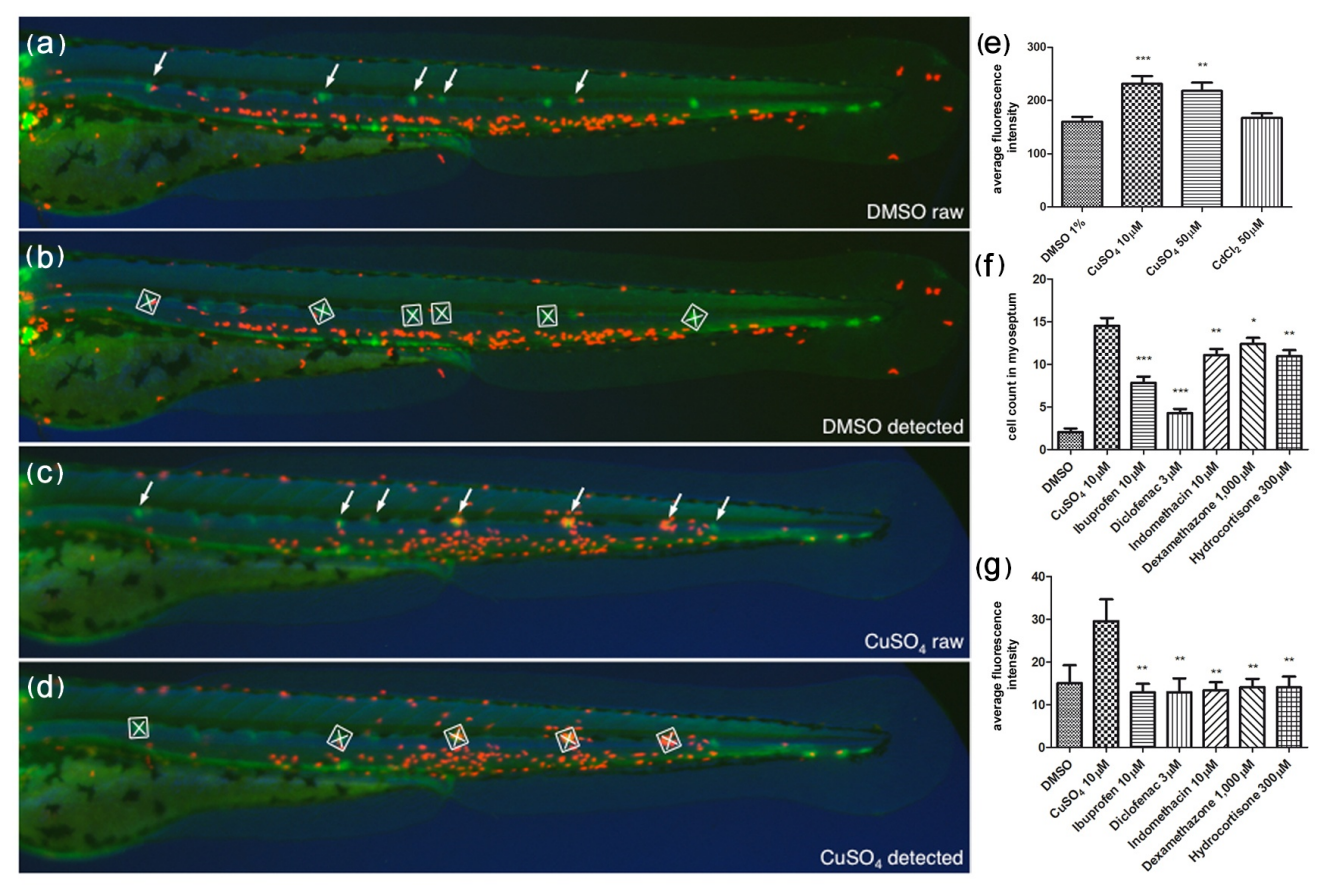
**Automated ChIn assay**. **(a-d) **Image acquisition method using compound transgenic larvae *cldnB::GFP *and *lysC::DsRED2*. Images show control (DMSO) **(a **and **b) **and treated (CuSO_4_) **(c **and **d) **fish revealing neuromasts (green, arrows) and leukocytes (red). Shown are the raw images **(a **and **c) **and the number and identity of the neuromasts that were automatically detected by the software **(b **and **d) **(white squares). The image analysis software determines the average red fluorescence intensity per square (neuromast area) and calculates the data averaged for all squares detected within one larva. Note that the program is able to detect most, but not all, of the visible neuromasts. The variable neuromast detection success is compensated by using more larvae than in the manual method: 24 per plate, in triplicate, averaging around 50 data-producing larvae per condition. **(e) **A control experiment using the automated ChIn assay. Untreated or metal-exposed double-transgenic fish were imaged, and red fluorescence was averaged from three experiments. Results are comparable to manual ChIn assays. **(f **and **g) **Comparison of ChIn assay results between the manual quantification method **(f) **and automated detection **(g) **of anti-inflammatory drug activity.

While manual quantification of migrating leukocytes using the ChIn assay is an improvement over other methods for analysis of selected molecules or for recovering mutations, it is still unrealistic as a method for high-throughput screening of hundreds to thousands of candidate chemicals or mutants with reasonable efforts and within reasonable time. Therefore, we sought to transform the ChIn assay into a scalable method that could be used in large-format screens. We developed a custom software script enabling us to map fluorescent expression domains in zebrafish larvae and combined it with automated microscopy. To put the automated system into operation, we used compound transgenic *cldnB::GFP/lysC::DsRED2 *larvae, as green fluorescent neuromasts facilitated computer-aided automatic examination of the area surrounding the damaged tissue for the presence of red fluorescent leukocytes (Figures 5a-5d and Additional file 3). The assay was carried out essentially as described previously, except that the larvae were distributed in 384-well plates and drugs or copper were added directly to the individual wells using multichannel pipettes. Larvae were preincubated with drugs for 30 minutes and then exposed to copper treatment. Forty minutes after addition of 10 μM CuSO_4_, plates were placed under a fluorescence microscope equipped with a ×2.5 lens and three channels per well were automatically captured (bright-field microscope, GFP and DsRED2). Images were then automatically processed using our custom script to first identify the neuromasts (GFP) and subsequently to quantify infiltrated leukocytes in their surrounding area by fluorescence intensity (DsRED2). To validate the automation of the ChIn assay, we first performed a control experiment testing different concentrations of CuSO_4 _and CdCl_2 _(Figure 5e). As with manual ChIn, the results of the automated assay revealed a significant increase of leukocytes in the vicinity of the neuromasts upon copper treatment as compared to control or cadmium treatment (compare Figures 2b and 5e). Furthermore, automated ChIn evaluation of a selected panel of drugs yielded results identical to those seen using the manual assay. The immunosuppressive effect for all compounds tested was detected with sensitivity comparable, or superior, to manual screening (compare Figures 5f and 5g). These results show that the ChIn assay provides sufficient robustness to be applied to automated detection and evaluation and will thus allow further upscaling by, for example, removing any remaining manual steps, including the introduction of robotic liquid handling.

## Discussion

We describe a new method, the ChIn assay, which will be of value for the initial detection of lead compounds exhibiting immunomodulatory activity as well as genes with roles in the different stages of the inflammatory response. In zebrafish, myeloid leukocytes develop during the first day of life, while molecular markers and cellular components of adaptive immunity arise well after the third day [23-25]. Thus, assays carried out in early larvae allow specific analysis of the innate immune system. These assays are facilitated by the transparency of the larvae and the availability of fluorescent tags in specific immune cell types in transgenic fish. However, nontransgenic zebrafish can also be used because histochemical stains (i.e., Sudan Black) are equally useful for ChIn assays.

The inflammatory response in this species has been well characterized [2,6,7,10,26]. However, studies carried out to date have relied on physical damage [9,10,27] or on a genetically induced chronic inflammatory condition [19] to observe the behavior of myeloid leukocytes. Our approach takes advantage of noninvasive tissue damage and induction of an acute inflammatory response in specific areas of the larva. The principal advantages of this method are its robustness and scalability: Physical manipulation of individual larvae is unnecessary, thus avoiding the introduction of variability and limitations to the number of individuals that can be screened. Using the ChIn assay, larvae are distributed singly into microtiter well plates, but can then be treated simultaneously with automated liquid-handling devices for induction of chemical damage, a procedure that is highly reproducible between individuals. The inflammatory reaction occurs within minutes. Thus, live treated larvae should be analyzed shortly after the addition of copper to the medium. Analysis of live transgenic animals allows for continuous or repeated monitoring of the immune cells' behavior, a useful condition for examining sequential arrival of different cell types or resolution of inflammation, for example. Alternatively, larvae can be fixed at the desired time point after treatment, allowing analysis of large sets of fish subjected to an identical treatment. In the latter case, the use of transgenic zebrafish lines is optional as it is possible to label leukocytes using histochemical stains, antibodies or *in situ *hybridization (Additional files 1 and 6). Nonetheless, fluorescent lines greatly facilitate the analysis and, in our hands, were essential for automation. We combined transgenic backgrounds that label the neuromasts in one color and immune cells in another (Figure 1r), which made the image recognition software highly efficient in selecting the area to analyze.

We applied several known anti-inflammatory drugs to test the ChIn assay. The selected molecules affect diverse pathways implicated in inflammation such as the COX, c-Jun N-terminal kinase (JNK) or peroxisome proliferator-activated receptor (PPAR)-γ responses. In addition, we confirmed that ROS gradient formation is critical for leukocyte recruitment upon copper-induced inflammation as chemical inhibition of NADPH oxidase significantly decreased the leukocyte recruitment toward neuromasts. Copper-mediated inflammation thus mechanistically recapitulates classical wounding assays without the need for manual manipulation of larvae. We caution that a primary large-scale, small-molecule screen carried out using the ChIn assay would not distinguish (1) a specific anti-inflammatory effect, (2) an inhibitory effect on general cell motility and (3) protection of hair cells from undergoing cell death, as all of these events would result in the same net effect: Recruitment of leukocytes to neuromasts would be altered. Candidate leads need to be subjected to secondary screens to distinguish the specific aspect of immune cell behavior that is affected.

Using the ChIn assay, we have obtained comparable results with two transgenic lines and two histochemical stains (Sudan Black and 3,3'-diaminobenzidine, or DAB). New tools currently under development that will allow the *in vivo *identification of distinct subpopulations of immune cells will further enhance the benefits of the ChIn assay in the near future. For example, preliminary results using *lck::GFP *transgenic larvae, a lymphocyte reporter line, showed no behavioral change in these cells after copper exposure, as expected given the role of these cells in adaptive immunity (CAD and MLA, unpublished work).

### Other types of screens feasible with the ChIn assay

A major application of the ChIn assay will be the identification of immunomodulatory activities of small molecules. However, we foresee additional applications using this strategy. First, mutant screens aimed at detecting genetic components of the inflammatory response will be facilitated with this approach. The high-throughput advantage of the ChIn assay can be used for efficiently screening mutagenized fish, as well as for gain of function screens or antisense screens using morpholino oligonucleotides. Genetic mutations or knockdowns can be identified, given that their phenotypic consequence regarding leukocyte behavior during inflammation lies within the sensitivity threshold of the ChIn assay. Here we provide proof of principle for the identification of mutations using *was *mutant zebrafish as an example. The *was *mutant larvae exhibited a significant reduction of leukocytes infiltrating the lateral line neuromasts upon copper treatment. On top of identifying homozygous mutants, the level of sensitivity of the ChIn assay even allowed for discrimination between heterozygous and homozygous larvae.

Second, the localized damage induced in the superficial neuromasts could be used as a model for inflammation mediated by infection. Most models of bacterial infection, for example, require injection of the pathogens to study the immune response [[28,29]; reviewed in [8]]. Larvae in which neuromasts have been damaged may be susceptible to infection through the sites of injury with waterborne pathogens, or likewise stimulated with pathogen-associated molecular patterns (PAMPs) such as lipopolysaccharides or viral nucleic acids added to the water. It may even be possible to distinguish the acute inflammatory response induced by wounding from that mediated by infectious agents using the ChIn assay in different transgenic backgrounds.

The highly specific migration of leukocytes to the neuromasts offers an excellent opportunity for a detailed examination of the molecular players involved in directed cell migration and immune cell homing. In this case, immune cells can be imaged *in vivo *before and during the generation of the attractive signal, allowing the monitoring of cell behavior during the key transition between random walk and chemotaxis. Likewise, the path followed by these cells can be observed in living tissue, as well as in the diffusion of signals through interstitial space, for example, using hydrogen peroxide reporters [2]. As opposed to tail wounding or sectioning, damage to the neuromast generates a focal point of chemoattractants with radial diffusion, which could provide a more adequate scenario for analysis of leukocyte homing.

Our laboratory is particularly interested in the regeneration of lateral line hair cells, a model for sensory regeneration in general. The contribution of the immune system to regeneration has been well documented, as has the potential impediment to regeneration caused by prolonged tissue inflammation and fibrosis. We believe that the ChIn assay can be used to investigate the molecular mechanisms of immune system involvement in regeneration. Whether the arrival of immune cells to damaged neuromasts promotes or inhibits regeneration of sensory hair cells is currently under study. Interestingly, time-lapse observation of a neuromast during copper-induced damage shows that patrolling neutrophils and macrophages (and possibly other cell types) provoke disorganization of the surviving cells, separating them from one another (Additional file 3). As migrating leukocytes contain and release matrix-degrading enzymes, such as Mmp9, that diminish adherence and tissue integrity, they could assist in the reconstruction of the damaged organ by facilitating the rearrangement, proliferation and differentiation of regenerating cells. Obviously, hair cell death or the regeneration process itself could be the subject of high-throughput screens (genetic or chemical), as has been done previously with damage induced in hair cells by neomycin [30-32]. Finally, molecules that protect neuromast hair cells against damage by copper would preclude immune cell migration to these organs. Pretreatment of fish with antioxidants protects hair cells from the effects of waterborne copper [12], and thus this type of activity could also be uncovered in a small-molecule screen using the ChIn assay.

## Conclusions

In conclusion, the ChIn assay represents a new tool that will contribute to the understanding of the pathways that lead to homing and migration of innate immune cells, as well as providing a discovery model for molecules that may yield new leads to therapeutic treatment of immune disorders.

## Methods

### Animals

Zebrafish were maintained and raised in our facility under recommended conditions [33]. The following strains of fish were used in this study: AB (wild type), *casper *[34], *was *[7], *cldnB::GFP *[16], *BACmpx::GFP *[5], *Brn3c::mGFP *[35], *Tg(lyz:EGFP)nz117 *and *Tg(lyz:DsRED2)nz50*, herein named *lysC::GFP *and *lysC::DsRED2 *[6]. All embryos were collected by natural spawning, staged according to Kimmel *et al. *[36] and raised at 28°C in E3 medium (5 mM NaCl, 0.17 mM KCl, 0.33 mM CaCl_2_, 0.33 mM MgSO_4_, and 0.1% methylene blue, equilibrated to pH 7.0) in Petri dishes, as described previously [37]. Embryonic and larval ages are expressed in hours postfertilization (hpf). All animals subjected to experimentation were anesthetized in MS-222 (tricaine; A5040; Sigma, Saint Louis, MO, USA), and procedures complied with the guidelines of the Animal Ethics Committees of the University of Chile and Karlsruhe Institute of Technology.

### Chemicals

A 10 mM stock solution of CuSO_4 _(copper II sulfate pentahydrate, catalog no. 102780; Merck, Darmstadt, Germany) was prepared daily in bidistilled water in a glass beaker until dissolved completely. Likewise, CdCl_2 _(CB236; Matheson, Coleman & Bell, Cincinnati, OH, USA) was prepared at a stock concentration of 10 mM. Additional chemicals were ZnSO_4 _(catalog no. ZI-1705; Winkler S.A., Santiago, Chile), NiSO_4 _(72280; Sigma) and AgNO_3 _(101512; Merck), neomycin (N1876; Sigma), 3,3'-dihexyloxacarbocyanine iodide, or DiOC_6_(3), was purchased from Interchim, Montluçon, France (FP-46764A) or AnaSpec, Fremont, CA, USA (catalog number 84715), DPI (D2926; Sigma), DMSO (317275; Merck), paraformaldehyde (catalog no. 1.04005.1000; Merck), Sudan Black (380B; Sigma), and Tween 20 (P5927; Sigma).

The following anti-inflammatory drugs were tested: from Cayman Chemical Co. (Ann Arbor, MI, USA), ibuprofen (70280), diclofenac (70680), aspirin (70260), indomethacin (70270), *trans*-resveratrol (70675), rosiglitazone (71740), mifepristone (10006317), sulindac (10004386) and SP600125 (10010466). From Sigma-Aldrich (St. Louis, MO, USA): dexomethazone (D1756) and hydrocortisone (H4001).

### Neuromast damage protocol and the basic ChIn assay (manual quantification)

Zebrafish larvae of the *BACmpx::GFP *and *lysC::DsRED2 *strains were grown in E3 medium in groups of 40-50 larvae per 10-cm Petri dish until 56 hpf. Only those larvae that spontaneously hatched were used for the assays as artificial enzymatic dechorionation often damages neuromasts, causing spontaneous inflammation. Any fish that appeared developmentally delayed or otherwise abnormal were also excluded from further analysis.

Selected larvae were transferred to six-well plates (M8562; Sigma) in a volume of 6 ml of E3 solution lacking methylene blue, and 15 larvae were added per well. Stock solutions of CuSO_4 _were added directly to the wells, and incubation was carried out for 40 minutes at 28°C. Larvae were then fixed by transferring them to 1.5-ml microfuge tubes and replacing the E3 medium with 4% paraformaldehyde prepared in phosphate-buffered saline (PBS) and incubating for 1 hour at room temperature. During fixation and subsequent handling, the tubes were kept in the dark to avoid bleaching or fading of the fluorescent protein signal. After fixation, larvae were washed three times for 5 minutes each in PBS-Tween20 with gentle agitation. Examination of fluorescent cells and counting was carried out within the next 48 hours after fixation using a Leica (Wetzlar, Germany) MZ-12 fluorescent stereoscope. Labeled cells were counted under fluorescent illumination within 10 cell diameters of the horizontal myoseptum between the first somite and the end of the tail (see Figure 2) on one side of each larva. All experiments were carried out with a minimum of 15 larvae for each condition, and counts were carried out by two observers. For neomycin treatments, *BACmpx::GFP *fish were incubated in the indicated concentration of antibiotic for 1 hour, and cell counts were done as before. In these experiments, we used 96-hpf fish, as neomycin kills only mature hair cells in lateral line neuromasts. We confirmed cell death in these fish by using hair cell-specific markers.

For Sudan Black staining, we used fish of the *casper *mutant strain [34], which lack pigmentation in the body. Larvae at 56 hpf were incubated as before (no metal and 10 mM CuSO_4_), fixed, washed and incubated for 20 minutes in 0.5 ml of Sudan Black staining reagent in batches of 30 larvae. Larvae were then washed three times in 70% ethanol at room temperature with mild rocking. Labeled cells were counted as before under bright-field illumination under a dissecting stereoscope. The *was *mutant fish were kept in E3 supplemented with propylthiouracil (from 24 hpf until fixation) to suppress pigmentation. The *was^+/- ^*fish were mated with *was^-/- ^*fish. Entire clutches were scored prior to genotyping, which was carried out as described previously [7]. AB zebrafish were used as wild-type controls.

### Drug assays

All drugs were prepared according to the manufacturer's instructions at a stock concentration of 10 mM by dissolving in 100% DMSO, which was previously purged by gaseous nitrogen for 2 minutes. Drugs were stored at -20°C until use and were diluted immediately prior to being added to larvae medium.

Drugs were added to the required concentration into the wells containing the experimental larvae in E3 containing 1% DMSO. Positive and negative control larvae were incubated only with 1% DMSO. Incubation with drugs was done for 1 hour prior to addition of CuSO_4_, which was added directly to the wells containing the experimental and positive control larvae. Incubation after copper addition continued for another 40 minutes before fixation in 4% paraformaldehyde for 1 hour at ambient temperature in the dark. Counting of leukocytes was carried out as before.

### Statistical treatment

Data are presented as mean values ± SEM. Statistical analysis was performed using GraphPad Prism version 5.00 for Windows software (GraphPad Software, La Jolla, CA, USA). The probability level for statistical significance was *P *< 0.05. All statistics regarding leukocyte migration were evaluated with unpaired *t*-tests with Welch's correction.

### Image processing

For imaging, larvae were anesthetized and mounted in 1.5% low melting point agarose (peqGOLD 35-2099; PEQLAB Biotechnologie, Erlangen, Germany) dissolved in E3. Photographs were taken with a Leica DFC 300-FX camera and Leica SPE confocal microscope (Leica Microsystems, Wetzlar, Germany) and processed with Adobe PhotoShop (San José, CA, USA), Zeiss Axiovision (Carl Zeiss Microimaging GmbH, Jena, Germany) and Image J (version 4.2, http://rsbweb.nih.gov/ij/index.html) software. For time lapse imaging, we used a Zeiss Axiovert 200 M microscope equipped with a ×20 lens objective and an Axiocam camera (Additional file 2) or a Leica SPE confocal microscope using a ×40 lens objective (Additional file 3). As described in Additional file 2 larvae were incubated in a 1:3,000 dilution of DiOC_6_(3) stock solution (1 mg/ml) in E3 and were then washed three times with E3 for 5 minutes. Larvae were embedded in 1.5% low melting point agarose dissolved in E3 containing 50 μM CuSO_4_. Images were captured every 90 seconds for a total of 150 minutes. As described in Additional file 3 compound transgenic fish (*cldnB::GFP*, *lysC::DsRED2*) were treated with 50 μM CuSO_4 _for 5 minutes and then mounted in 1.5% low melting point agarose dissolved in E3 containing 50 μM CuSO_4_. Images were captured every 60 seconds for a total of 100 minutes.

### Automated ChIn assay

Individual anesthetized larvae were manually placed in single wells of a 384-well plate in embryo buffer. Subsequently, compound stock solution in DMSO was transferred from a compound source plate to the assay plate containing embryo buffer (E3 + 1% DMSO + 0.02% MS222) and larvae using a multichannel pipette. The assay plate containing larvae and compound was sealed and incubated for 30 minutes at 28°C. Following compound incubation, CuSO_4 _solution (0.3 mM) was added to the assay plate, resulting in a final concentration of 10 μM copper sulfate. Assay plates were then incubated for another 40 minutes at 28°C. This procedure yielded a total volume of 120 μl per well and resulted in the respective final compound screening concentrations for this assay (10 μM ibuprofen, 3 μM diclofenac, 10 μM indomethacin, 1 mM dexamethazone, 300 μM hydrocortisone). A volume of 120 μl was empirically identified as the optimal volume for our automated imaging procedure. To achieve an assay sensitivity comparable to manual analysis, we also defined the minimum number of individual larvae analyzed per condition to be 30. In practice, 24 embryos for one condition were imaged per 384-well plate, but experiments were repeated at least in triplicate such that, on average, 50 larvae were analyzed per condition (see below).

Automatic imaging was performed on Olympus Scan^R high-content screening microscope setups (Olympus Biosystems, Munich, Germany) equipped with a ×2.5 lens objective (plan-apochromatic), an Olympus Biosystems DB-1 digital camera (1,300 × 1,024 pixels), filter cubes for GFP excitation filter, 460-480 nm; emission filter, 495-540 nm; dichromatic mirror, 485 nm) and cyanine 3 (Cy3) (excitation filter, 535-555 nm; emission filter, 570-625 nm; dichromatic mirror, 565 nm), and an ultrastable light source MT-20 xenon lamp. Camera image integration times were fixed (20-ms bright-field microscope, 400-ms GFP, 150-ms Cy3). An object detection autofocus algorithm detected the central focal plane of the first-well larva and was applied for the rest of the plate. Image processing was done using the LabView Vision AI rapid prototyping tool (National Instruments, Munich, Germany). Data management, red-green-blue (or RGB) overlay gallery generation and result display were performed using self-made LabView software modules. These scripts enable the detection of GFP-labeled neuromasts on an extended focus projection of five optical sections (two in each direction from the central focal plane) and define an empirically established surrounding area (see Figure 4) in the GFP channel. Subsequently, the inflammatory response is quantified by the detection of DsRED2-labeled leukocytes within the area surrounding the neuromasts in the red channel. Quantification is based on the average relative fluorescence intensity of leukocytes within this area. For proper image processing, the larvae ideally have to be oriented in a lateral position. However, our image analysis scripts allowed a certain degree of freedom with regard to this requirement. Our procedures yielded more than 70% of larvae positioned in a way that allowed automated image processing without the need for manual orientation of the larvae. However, all plates were manually checked, and larvae in unfavorable positions were corrected. All modules are available on request from the authors.

## Authors' contributions

CD and OP performed all of the manual ChIn assays and statistical analysis and prepared the figures. CW and CG did experiments related to automated ChIn and Sudan Black stains on *was *mutant fish. VG discovered copper-induced expression of *mmp9 *and carried out *in situ *hybridization. RJ generated the adult homozygous *was *mutant fish. FL, UL and CG worked on the automated screening system and generated the corresponding data and figures. MA conceived the idea and wrote the initial draft of the paper, which was edited by CG. All authors read and approved the final manuscript.

## Supplementary Material

Additional file 1 **Supplementary Figure 1. Induction of *matrix metalloproteinase 9 *(*mmp9*) expression by copper treatment in zebrafish larvae**. *In situ *hybridization to detect expression of *mmp9 *was carried out in control **(a **and **c) **and sibling fish treated with copper sulfate **(c **and **d)**. **(a **and **b) **Induction of *mmp9 *expression after treating 2-day-old fish with 10 μM CuSO_4 _for 40 minutes. While control fish have few detectable cells labeled with probe, clusters of highly labeled cells are seen in a characteristic pattern along the flanks of the treated animals. The *inset *shows a closeup image of one of these clusters. **(c **and **d) ***mmp9 *induction by treatment of 3-day-old fish with 100 μM CuSO_4 _for 6 hours and fixed immediately thereafter. Note that clustering of labeled cells at discrete positions along the midline is also apparent after the more severe treatment. Click here for file

Additional file 2 **Supplementary Movie 1. Migration of leukocytes toward damaged neuromasts**. Transgenic *BACmpx::GFP *larvae were stained with 3,3'-dihexyloxacarbocyanine iodide (DiOC_6_) to reveal neuromasts (red arrows; individual labeled cells on the skin are chemosensory cells). In the movie, the trunk and part of the tail are shown anterior to the left. The images are displayed without pseudocoloring, given that DiOC_6 _is visible in the green fluorescent protein (GFP) fluorescence channel. After adding 50 μM CuSO_4 _to the medium, fish were mounted and immediately imaged for 2 hours to observe the behavior of leukocytes. Two GFP-labeled cells have been colored red to follow their trajectories toward the nearest neuromast. A wavelike contraction of the neuromast can be seen near the beginning of the sequence. Note the large number of leukocytes that arrive at the posterior-most neuromast after copper damage. Original magnification, ×20. Click here for file

Additional file 3 **Supplementary Movie 2. Leukocytes patrol among neuromast cells after copper induced damage**. Description: Compound *cldnB::GFP*, *lysC::DsRED2 *transgenic fish that have green-labeled neuromasts and red-labeled leukocytes were treated with 50 μM CuSO_4 _and immediately mounted for imaging for 1 hour under a confocal microscope. Images were taken in both channels and in bright-field illumination every 60 seconds in three *z*-planes for 1 hour and were then combined for every time point to produce the movie. Note the progressive disorganization of the neuromast cells, which loses its rosette structure while cells become detached from one another and leukocytes actively migrate throughout the organ. Original magnification, ×40. Click here for file

Additional file 4 **Supplementary Figure 2. Neutrophils and macrophages behave similarly in response to copper exposure**. Compound *BACmpx::GFP/lysC::DsRED2 *transgenic fish were treated with 10 μM copper sulphate, and the area surrounding a neuromast was imaged 20 minutes after initiation of exposure. Detection of cells was carried out in the GFP channel **(a) **and the red (DsRED2) channel **(b)**, and both images were merged **(c)**. Both neutrophils (yellow cells in **(c)**) and macrophages (red cells in **(c)**) can be observed to migrate to damaged neuromasts. Click here for file

Additional file 5 **Supplementary Figure 3. Behavior of leukocytes after long-term copper exposure in zebrafish larvae**. At 3 days postfertilization (dpf), transgenic *lysC::DsRED2 *fish were left untreated **(a, c, e**, and **g) **or exposed permanently thereafter to 10 μM CuSO_4 _**(b, d, f, h**, and **i-k) **and were imaged daily until 7 dpf (times in the right-hand column are expressed in hours posttreatment, hpt). **(a-h) **Lateral views of entire larvae. Note the general dispersal of leukocytes in treated vs. control fish simultaneous with accumulation in different anterior regions, especially the branchial arches of the animal beginning 1 day after beginning treatment. **(i-k) **Closeups of specific areas at 72 hpt. **(i) **Ventral view of branchial arches. **(j) **Lateral view of head; arrow indicates olfactory pit area. Closeup view of the area surrounding a neuromast. Note that fish exposed for long periods to copper sulfate suffer developmental delays. Click here for file

Additional file 6 **Supplementary Figure 4. Chemically induced inflammation assay (ChIn) using Sudan Black**. (**a **and **b**) Bright-field images of untreated **(a) **and 10 μM CuSO_4_-treated **(b) **56-hpf *casper *larvae stained with Sudan Black to reveal leukocytes. Note the congregation of labeled cells at the posterior lateral line neuromasts (arrows). **(c) **Quantification of leukocyte migration (detected by Sudan Black staining) to the lateral line in untreated and metal-exposed larvae. The result is equivalent to that obtained with *BACmpx::GFP *or *lysC::DsRED2 *transgenic larvae. Click here for file

Additional file 7 **Supplementary Figure 5. Neomycin ablates hair cells but fails to induce localization of leukocytes to the horizontal myoseptum**. **(a **and **c) **Transgenic *Brn3c::mGFP *larvae express GFP in hair cells of the lateral line neuromasts as well as in cells of the ear, eye and brain. Transgenic larvae were left untreated **(a) **or were treated with 100 μM neomycin for 2 hours **(b) **and imaged under fluorescence. Note the ablation of lateral line hair cells, though other expressing tissues are unaffected. **(b **and **d) ***BACmpx::GFP *larvae were treated in parallel with *Brn3c::GFP *larvae to examine leukocyte behavior. Note that while leukocytes disperse with the neomycin treatment, they do not congregate near neuromasts as they do with copper treatment. Click here for file

Additional file 8 **Supplementary Figure 6. The ChIn assay can be adapted to carry out an inflammation resolution screen**. *BACmpx::GFP *transgenic fish were raised and treated at 56 hpf with 10 μM CuSO_4 _as described for the ChIn assay. The number of infiltrating leukocytes in the lateral line were counted and compared to those of untreated fish at 0 hours posttreatment (hpt). These fish were then left for a further 3 hours after removal of copper (3 hpt) and examined once again to count infiltrating leukocytes. Copper-treated fish show significant diminishment of leukocyte numbers by this time, indicating resolution of inflammation. If copper-treated fish are exposed to diclofenac or ibuprofen at 0 hpt and examined at 3 hpt, the number of infiltrating leukocytes remains high, indicating that these drugs inhibit resolution. Click here for file
