# Supplementary figures and images for: A high-throughput chemically induced inflammation assay in zebrafish
Source: BMC Biol. 2010 Dec 22;8:151. doi: 10.1186/1741-7007-8-151 (PMC3022775; doi:10.1186/1741-7007-8-151)

58hpf

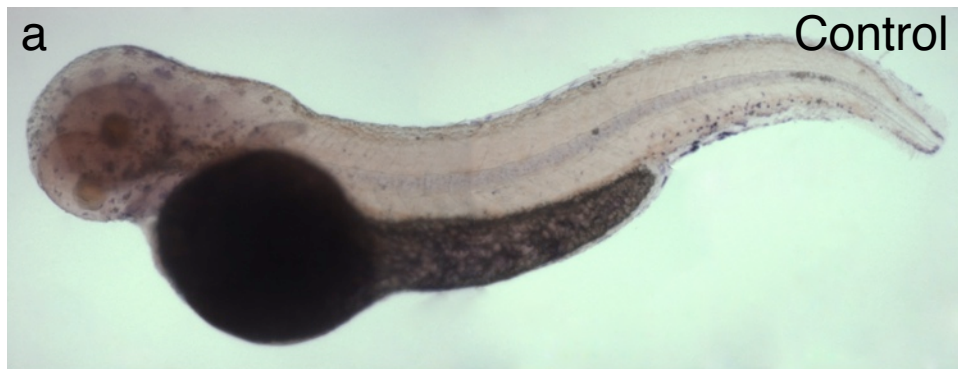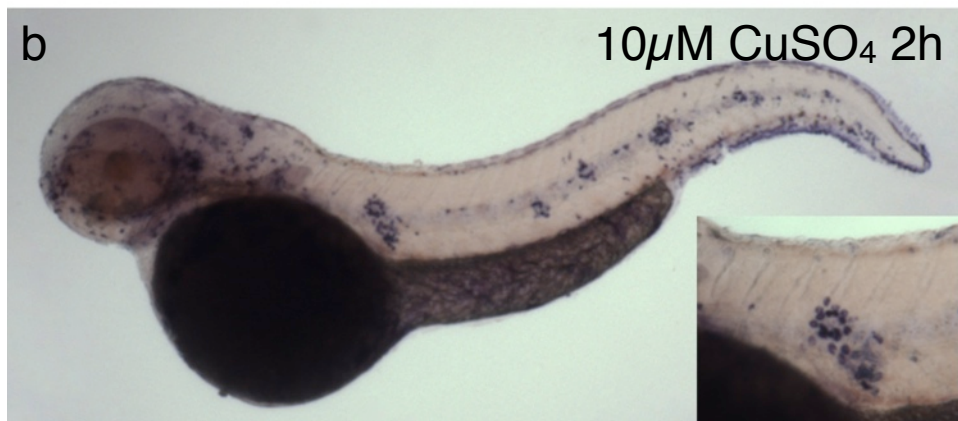

78hpf

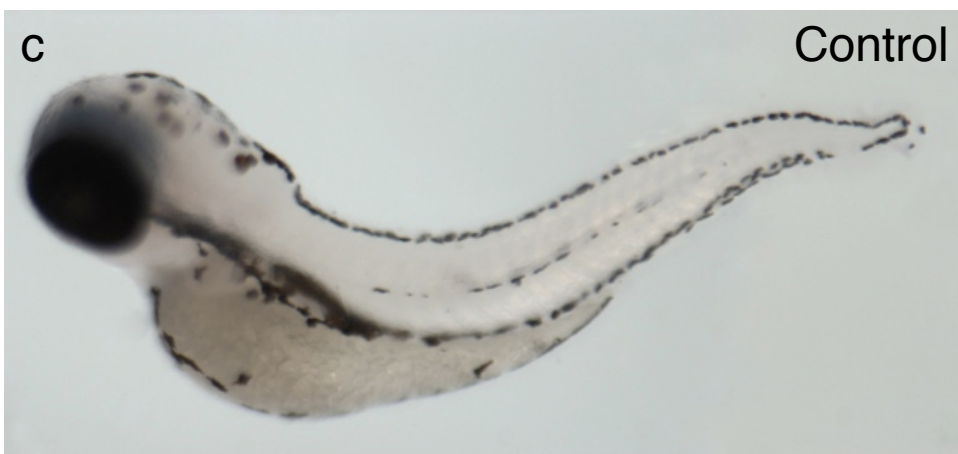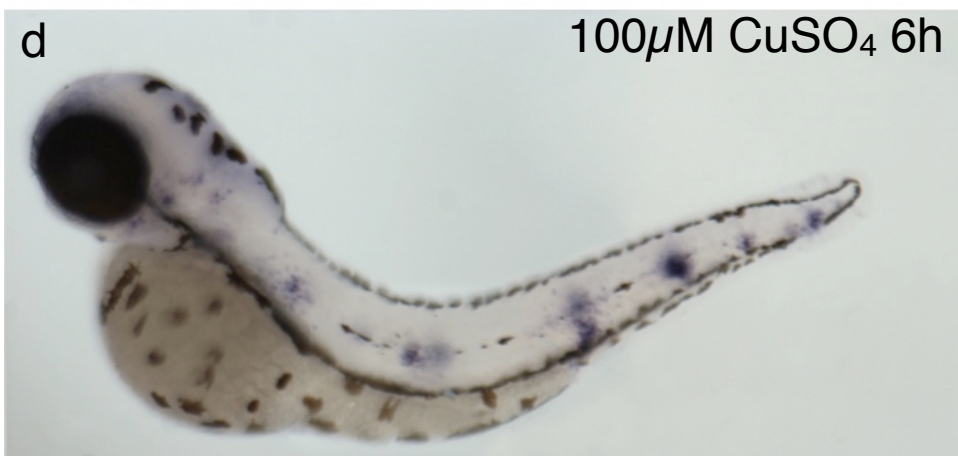

Supplement: Additional file 1 — Supplementary Figure 1. Induction of matrix metalloproteinase 9 (mmp9) expression by copper treatment in zebrafish larvae. In situ hybridization to detect expression of mmp9 was carried out in control (a and c) and sibling fish treated with copper sulfate (c and d). (a and b) Induction of mmp9 expression after treating 2-day-old fish with 10 μM CuSO4 for 40 minutes. While control fish have few detectable cells labeled with probe, clusters of highly labeled cells are seen in a characteristic pattern along the flanks of the treated animals. The inset shows a closeup image of one of these clusters. (c and d) mmp9 induction by treatment of 3-day-old fish with 100 μM CuSO4 for 6 hours and fixed immediately thereafter. Note that clustering of labeled cells at discrete positions along the midline is also apparent after the more severe treatment. [file 1741-7007-8-151-S1.pdf]

a

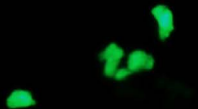

*BACmpx::GFP*

b

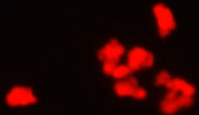

*lysC::DsRED2*

c

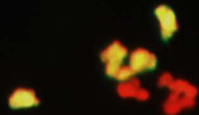

*BACmpx::GFP*  
*lysC::DsRED2*

Supplement: Additional file 4 — Supplementary Figure 2. Neutrophils and macrophages behave similarly in response to copper exposure. Compound BACmpx::GFP/lysC::DsRED2 transgenic fish were treated with 10 μM copper sulphate, and the area surrounding a neuromast was imaged 20 minutes after initiation of exposure. Detection of cells was carried out in the GFP channel (a) and the red (DsRED2) channel (b), and both images were merged (c). Both neutrophils (yellow cells in (c)) and macrophages (red cells in (c)) can be observed to migrate to damaged neuromasts. [file 1741-7007-8-151-S4.pdf]

## Control

## CuSO<sub>4</sub>

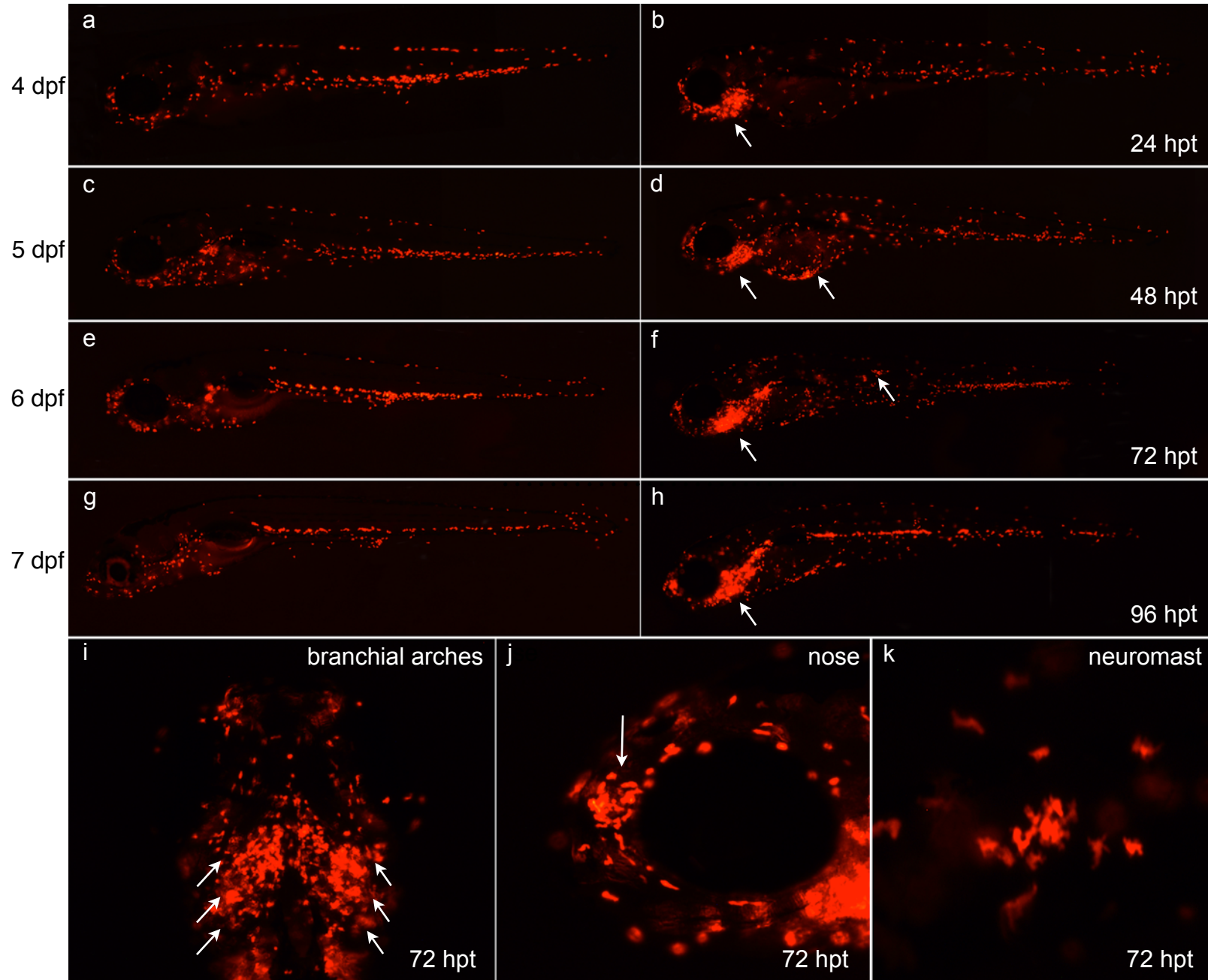

Supplement: Additional file 5 — Supplementary Figure 3. Behavior of leukocytes after long-term copper exposure in zebrafish larvae. At 3 days postfertilization (dpf), transgenic lysC::DsRED2 fish were left untreated (a, c, e, and g) or exposed permanently thereafter to 10 μM CuSO4 (b, d, f, h, and i-k) and were imaged daily until 7 dpf (times in the right-hand column are expressed in hours posttreatment, hpt). (a-h) Lateral views of entire larvae. Note the general dispersal of leukocytes in treated vs. control fish simultaneous with accumulation in different anterior regions, especially the branchial arches of the animal beginning 1 day after beginning treatment. (i-k) Closeups of specific areas at 72 hpt. (i) Ventral view of branchial arches. (j) Lateral view of head; arrow indicates olfactory pit area. Closeup view of the area surrounding a neuromast. Note that fish exposed for long periods to copper sulfate suffer developmental delays. [file 1741-7007-8-151-S5.pdf]

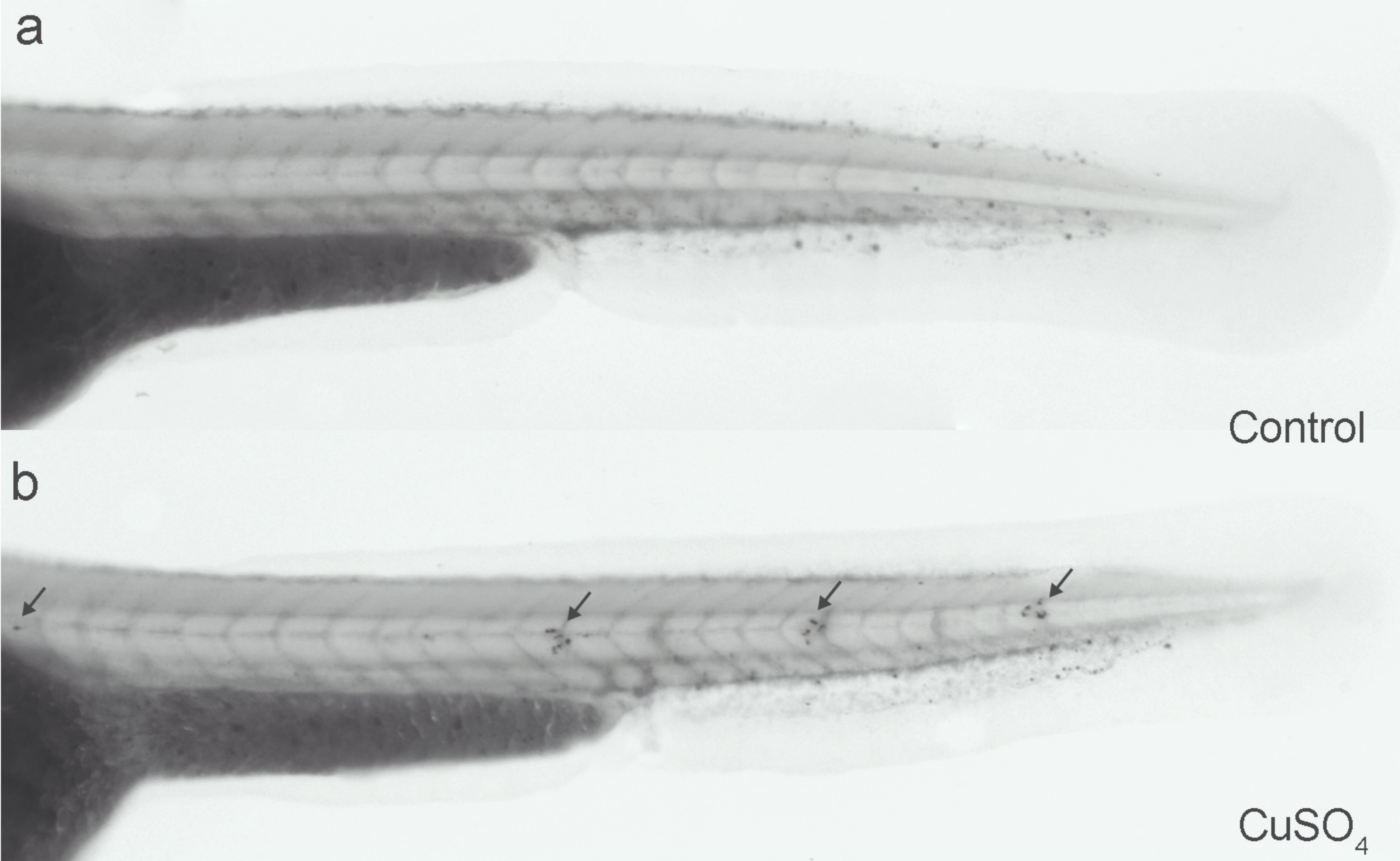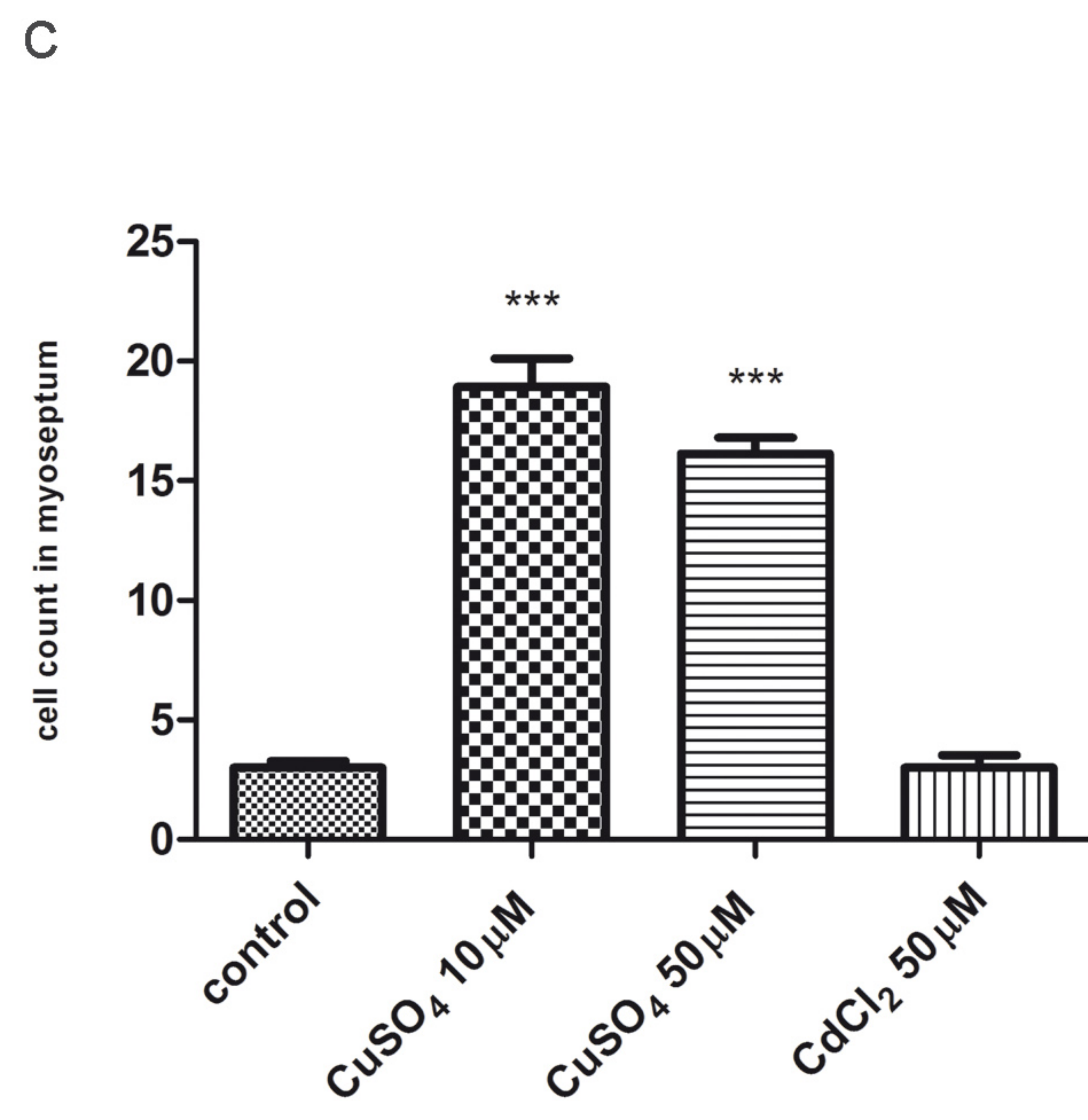

Supplement: Additional file 6 — Supplementary Figure 4. Chemically induced inflammation assay (ChIn) using Sudan Black. (a and b) Bright-field images of untreated (a) and 10 μM CuSO4-treated (b) 56-hpf casper larvae stained with Sudan Black to reveal leukocytes. Note the congregation of labeled cells at the posterior lateral line neuromasts (arrows). (c) Quantification of leukocyte migration (detected by Sudan Black staining) to the lateral line in untreated and metal-exposed larvae. The result is equivalent to that obtained with BACmpx::GFP or lysC::DsRED2 transgenic larvae. [file 1741-7007-8-151-S6.pdf]

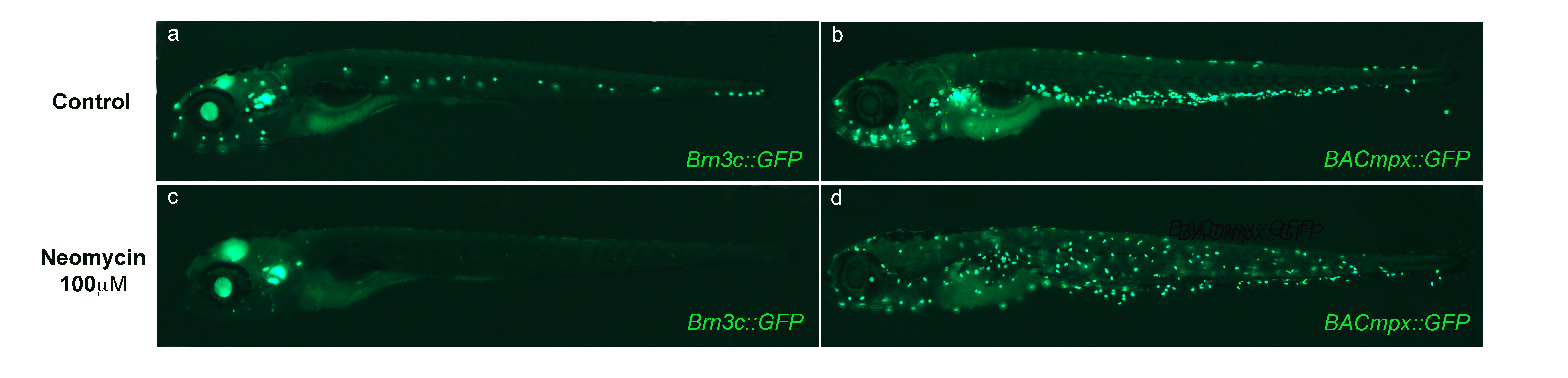

Supplement: Additional file 7 — Supplementary Figure 5. Neomycin ablates hair cells but fails to induce localization of leukocytes to the horizontal myoseptum. (a and c) Transgenic Brn3c::mGFP larvae express GFP in hair cells of the lateral line neuromasts as well as in cells of the ear, eye and brain. Transgenic larvae were left untreated (a) or were treated with 100 μM neomycin for 2 hours (b) and imaged under fluorescence. Note the ablation of lateral line hair cells, though other expressing tissues are unaffected. (b and d) BACmpx::GFP larvae were treated in parallel with Brn3c::GFP larvae to examine leukocyte behavior. Note that while leukocytes disperse with the neomycin treatment, they do not congregate near neuromasts as they do with copper treatment. [file 1741-7007-8-151-S7.jpeg]

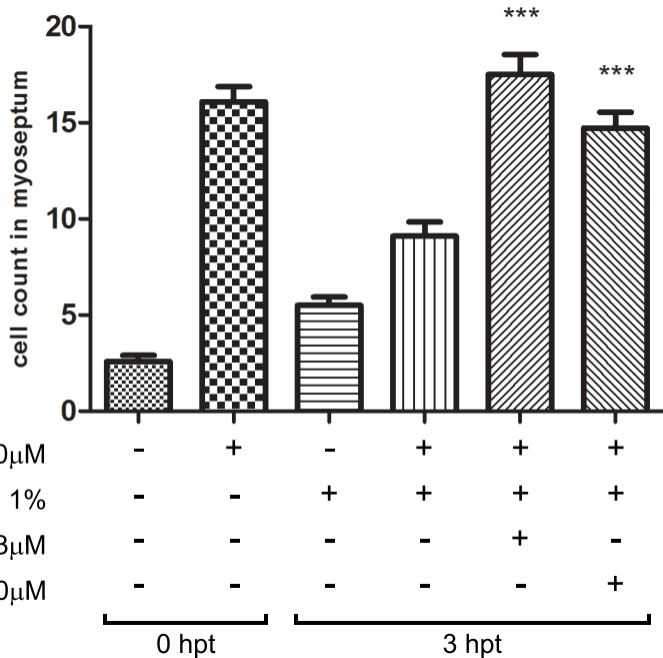

CuSO<sub>4</sub> 10μM

-

+

DMSO 1%

-

+

Diclofenac 3μM

-

-

+

Ibuprofen 10μM

-

-

-

+

0 hpt

3 hpt

Supplement: Additional file 8 — Supplementary Figure 6. The ChIn assay can be adapted to carry out an inflammation resolution screen. BACmpx::GFP transgenic fish were raised and treated at 56 hpf with 10 μM CuSO4 as described for the ChIn assay. The number of infiltrating leukocytes in the lateral line were counted and compared to those of untreated fish at 0 hours posttreatment (hpt). These fish were then left for a further 3 hours after removal of copper (3 hpt) and examined once again to count infiltrating leukocytes. Copper-treated fish show significant diminishment of leukocyte numbers by this time, indicating resolution of inflammation. If copper-treated fish are exposed to diclofenac or ibuprofen at 0 hpt and examined at 3 hpt, the number of infiltrating leukocytes remains high, indicating that these drugs inhibit resolution. [file 1741-7007-8-151-S8.pdf]
